# BCL-2 as therapeutic target for hematological malignancies

**DOI:** 10.1186/s13045-018-0608-2

**Published:** 2018-05-11

**Authors:** Guilherme Fleury Perini, Glaciano Nogueira Ribeiro, Jorge Vaz Pinto Neto, Laura Tojeiro Campos, Nelson Hamerschlak

**Affiliations:** 1 Hospital Israelita Albert Einstein, Av. Albert Einstein, 627, Sao Paulo, Sao Paulo 05652-900 Brazil; 2 Hospital das Clínicas da Universidade Federal de Minas Gerais, Av. Prof. Alfredo Balena, 110, Santa Efigênia, Belo Horizonte, Minas Gerais 30130-100 Brazil; 3 Cettro-Centro de Câncer de Brasília, SMHN Quadra 2, Bloco A, Edifício de Clínicas, 12 andar, Brasília, DF 70710-904 Brazil; 4 AbbVie, Avenida Jornalista Roberto Marinho, 85-7 andar, Brooklin, Sao Paulo, Sao Paulo 04576-010 Brazil

**Keywords:** Apoptosis, Resistance, BCL-2, BH3-mimetics, Venetoclax, Leukemia, Lymphoma, Hematological malignancies

## Abstract

Disruption of the physiologic balance between cell proliferation and cell death is an important step of cancer development. Increased resistance to apoptosis is a key oncogenic mechanism in several hematological malignancies and, in many cases, especially in lymphoid neoplasias, has been attributed to the upregulation of BCL-2. The BCL-2 protein is the founding member of the BCL-2 family of apoptosis regulators and was the first apoptosis modulator to be associated with cancer. The recognition of the important role played by BCL-2 for cancer development and resistance to treatment made it a relevant target for therapy for many diseases, including solid tumors and hematological neoplasias. Among the different strategies that have been developed to inhibit BCL-2, BH3-mimetics have emerged as a novel class of compounds with favorable results in different clinical settings, including chronic lymphocytic leukemia (CLL). In April 2016, the first inhibitor of BCL-2, venetoclax, was approved by the US Food and Drug Administration for the treatment of patients with CLL who have 17p deletion and had received at least one prior therapy. This review focuses on the relevance of BCL-2 for apoptosis modulation at the mitochondrial level, its potential as therapeutic target for hematological malignancies, and the results obtained with selective inhibitors belonging to the BH3-mimetics, especially venetoclax used in monotherapy or in combination with other agents.

## Background

Resistance to apoptosis is an important hallmark of cancer, contributing to clonal cell development, tumor growth, and resistance to treatment [1]. In addition, blockage of apoptosis may also lead to the accumulation of other oncogenic alterations, including uncontrolled proliferation. Over the last decades, a remarkable progress in cancer research and therapy has been achieved, with a better understanding of the apoptotic machinery and the association between tumors and altered expression of proteins involved in apoptosis regulation [2, 3]. The members of the B-cell lymphoma-2 (BCL-2) family are central regulators of apoptosis triggered by environmental cues and in response to multiple stress signals [4]. In many cancers, high levels of anti-apoptotic proteins such as BCL-2 and BCL-X_L_ were shown to contribute not only to tumor initiation and progression, but also to lack of response to chemotherapy [5, 6].

*BCL-2* was the first gene shown to promote prolonged cell survival rather than increased proliferation [4, 7]. This discovery led to the concept that inhibition of apoptosis is an important step in tumorigenesis [4]. Promising results are being reported with the use of inhibitors of BCL-2 and other related molecules, especially with BH3-mimetics [8, 9]. Given that apoptosis blockage is a key oncogenic mechanism in lymphoid malignancies, and that BCL-2 overexpression is a common finding in leukemias and lymphomas, many antagonists of anti-apoptotic BCL-2 have been developed and investigated for the treatment of hematological neoplasms [2, 6].

BH3-mimetics comprise a novel class of BCL-2 inhibitors that have shown promising results in several hematological malignancies, both as single agents and in combination with other anti-cancer drugs. Among the BH3-mimetics, venetoclax (also known as ABT-199), a potent and selective inhibitor of BCL-2, was recently approved by the US Food and Drug Administration (FDA) for the treatment of relapsed/refractory chronic lymphocytic leukemia (CLL) with 17p deletion based on its favorable safety profile and high response rates [10]. Here, we review the role of BCL-2 protein on apoptosis regulation, its importance as therapeutic target for hematological malignancies and the results obtained with BH3-mimetics drugs on preclinical and clinical trials.

## The apoptosis machinery

Apoptosis is a highly complex and well-regulated form of programmed cell death. It plays an essential role in embryogenesis, tissue development, immunity, and maintenance of homeostasis. However, both excessive and insufficient cell death can lead to a wide variety of pathological conditions including neurodegenerative diseases, immunological disorders, and cancer [11–13]. In the hematopoietic system, programmed cell death exerts an important role, allowing cell turnover and rapid expansion and retraction of cell populations in response to infection [14].

Caspase activation plays a crucial role in apoptosis, with caspases being known as the central executioners of the apoptotic machinery. The proteolytic events mediated by caspases result in peculiar morphological and ultrastructural changes in dying cell that, ultimately, define the apoptotic phenotype [15]. Upon activation, caspases can often cleave and activate other procaspases, initiating a proteolytic cascade. In addition, some procaspases are also capable to form aggregates and undergo autoactivation. This proteolytic cascade, in which one caspase can activate other caspases, and in some cases, activate themselves, allows the amplification of signaling that leads to cell death [16].

Two major pathways for caspase activation and apoptosis initiation have been described in vertebrates: the extrinsic pathway and the intrinsic pathway. The extrinsic pathway involves the activation of cell death receptors located on the cell surface, such as tumor necrosis factor receptors or Fas, and whose interaction to their ligands promote the activation of caspase-8. In the intrinsic pathway, also known as the mitochondrial pathway, disruption of mitochondrial integrity is the crucial decision point [17]. Mitochondria outer membrane permeabilization (MOMP) allows proteins located in the intermembrane space of the mitochondria to be released into the cytosol, thus triggering apoptosis [4].

Both intrinsic and extrinsic pathways culminate on the activation of a caspase cascade that will activate the signaling route leading to the morphological features that characterize apoptotic cells. Cell shrinkage, plasma membrane blebbing with no loss of integrity, DNA fragmentation, and condensation of chromatin are among the characteristics exhibited by cells undergoing apoptosis. This culminates with the formation of apoptotic bodies and their subsequent rapid removal by phagocytosis and degradation by other surrounding cells [11, 16]. The apoptotic phenotype is elicited by the cleavage of cellular substrates by caspases, especially activated caspase-3 [16].

Apoptosis can be triggered by a variety of physiologic and pathologic stimuli. The mitochondrial pathway is activated by different forms of cellular stress, such as DNA damage and loss of survival signals [8]. It is also the apoptotic pathway activated by most cytokine deprivation, chemicals, and by therapeutic agents, including cancer chemotherapeutic drugs and irradiation [18]. The mitochondria play a central role in apoptosis regulation and while caspases are referred to as the executioners of apoptosis, the BCL-2 family members are known as the regulators, given their central role in controlling the integrity of the outer mitochondrial membrane [4, 17].

## The BCL-2 family

The BCL-2 family comprises both pro-apoptotic and anti-apoptotic members [4]. Regarding their structure and function, members of this family can be grouped in three main categories. The anti-apoptotic subfamily is characterized by the presence of four BCL-2 homology (BH) domains (BH1, BH2, BH3, and BH4) and, in humans, includes the proteins BCL-2 (the founding member), BCL-X_L_, BCL-w, BCL-2-related protein A1 (Bfl-1/A1), myeloid cell leukemia 1 (MCL-1), and BCL-B/Boo. The pro-apoptotic members can be divided in two subfamilies: the multi-domain pro-apoptotic ‘effectors’ (such as BAK and BAX) and those members known as ‘BH3-only proteins’ as they only have the short BH3 domain. The latter subfamily includes BAD, BID, BIK, BIM, BMF, HRK, PUMA, and NOXA [4, 19].

Activator BH3-only proteins such as BID or BIM are activated by transcriptional or post-translational modification in response to cellular damage or deregulation, and activate effectors such as BAX and BAK, which in turn are responsible for initiating the mitochondrial permeabilization and cell death processes. Although BAX and BAK were first believed to lack the BH4 domain, this domain was later identified in these proteins [6].

Anti-apoptotic members of the BCL-2 family prevent MOMP through two mechanisms: the binding and sequestration of the active forms of BAK and BAX, and/or the sequestration of the direct activators BH3-only proteins. By binding to BAX and BAK, anti-apoptotic proteins such as BCL-2 prevent them from oligomerizing [6].

BH3-only proteins modulate the activity of the other two groups of BCL-2 proteins [9, 20]. They can initiate apoptosis either by inhibiting the anti-apoptotic BCL-2 family members or by directly activating pro-apoptotic family proteins [20]. BH3-only proteins can circumvent the inhibitory effect of the anti-apoptotic BCL-2 family members by promoting the indirect activation of BAX and BAK, as they bind through their α-helical BH3 motif to the hydrophobic groove of anti-apoptotic proteins, releasing BAX and BAK from the sequestration, and enabling BAK and BAX to oligomerize within the mitochondrial outer membrane [6, 12, 17]. Importantly, due to structural differences, interactions between BH3-only proteins and anti-apoptotic proteins occur in a selective manner and with different affinities, with each BH3-only protein being able to bind to a subset or to all anti-apoptotic members [21]. In this respect, BIM, PUMA, and tBID (formed as result of the activation of the extrinsic pathway and cleavage of BID) can bind all anti-apoptotic BCL-2 proteins, whereas other members have a more restricted affinity [6, 21].

Mitochondria permeabilization can also be promoted through the direct activation of proapoptotic effectors by molecules like BID and BIM, the so-called direct activators [9, 17].

Mitochondria disruption in response to death signals results in the release of cytochrome c and other pro-apoptotic factors into the cytosol, which leads to the activation of the caspase cascade and apoptosis [4]. Deregulation of BCL-2 family members’ expression is observed in a variety of cancers, including hematopoietic neoplasms and solid tumors [2, 22]. Studying the copy-number profiles from 3131 cancer specimens of 26 distinct histological types, Beroukhim et al. found that anti-apoptotic genes of the *BCL-2* family were among the genes most commonly found in amplification peaks, while other pro-apoptotic genes were frequently found in deletion peaks [23].

## The BCL-2 protein

### Structure and function

Located on chromosome 18q21.33, the *BCL-2* gene was first discovered by cloning the breakpoint region of the t(14;18) translocation, a chromosomal abnormality commonly found in follicular lymphomas (FL). As a result of this translocation, the *BCL-2* gene at 18q21 is placed into juxtaposition with the immunoglobulin heavy chain locus region at 14q32 [5].

BCL-2 is a 26 kDa protein, located on the outer membrane of the mitochondria, but also found on the ER membrane and the nuclear envelope [4]. Although it shares structural similarities with BCL-X_L_, important differences were observed in healthy cells regarding the mechanisms by which BAK and BAX are restrained. BCL-X_L_ has been shown to sequester both BAX and BAK, while BCL-2 was reported to constrain only BAX, while being unable to prevent BAK activation [24].

Despite completing the embryonic development, BCL-2-deficient mice were shown to present massive lymphoid apoptosis after birth, renal failure due to polycystic kidney disease, and hypopigmented hair [25]. Initial analyses of the distribution of BCL-2 protein revealed that BCL-2 is expressed by various normal tissues and hematopoietic lineages, including the lymphocytes at germinal centers of the follicular mantle zone, regions related to maintenance of plasma cells and memory B cells, and T cells in the thymic medulla. The BCL-2 protein is also present in non-lymphoid tissues such as the epithelium and in long-lived postmitotic cells, such as neurons [26]. Later, BCL-2 expression in the nervous system was confirmed and shown to be important for neuronal survival, preventing apoptosis induced by nerve growth factor deprivation [27].

Vaux et al. showed that BCL-2 expression induced hematopoietic cell survival even in the absence of interleukin 3 and cooperates with MYC to induce proliferation of B-cell precursors [7]. BCL-2 synergizes with MYC in tumor progression, as observed in transgenic mice [28], and this cooperation seems to involve the capacity of BCL-2 to block MYC-induced apoptosis in lymphoid cells, without affecting the mitogenic function of MYC [29].

Graninger et al. reported that BCL-2, in addition to being expressed in leukemic cells, is expressed in a variety of normal hematopoietic lineages, including T cells, with levels of expression varying according to the cell population, stage of maturation, and activation. In normal B-cells, levels of BCL-2 mRNA were found to be upregulated in pre-B cell lines, but to decrease with differentiation towards a mature B-cell stage. Opposed to this, mature B-cell lymphomas bearing the translocation t(14;18) exhibit much higher levels of BCL-2 mRNA than matched counterparts in which the translocation is absent. As observed for other genes involved in tumorigenesis, BCL-2 levels were virtually undetectable in resting B cells, while they were augmented upon cell activation [30].

The oncogenic potential of BCL-2 was first demonstrated by Reed et al. in 1988 using gene transfer technology [31]. In the following year, BCL-2 gene rearrangements were described to be associated with poor prognosis in large-cell non-Hodgkin lymphomas (NHL) [32]. In 1990, Reed et al. showed that suppression of BCL-2 expression using antisense RNAs rendered leukemia cells susceptible to death by apoptosis [33]. As a result, over the last years, there was an explosive output of research focused on the role of BCL-2 and its family members in apoptosis’ regulation, cancer development, and resistance to treatment.

### BCL-2 overexpression in hematological malignancies

High levels of BCL-2 are observed in patients with FL, CLL, mantle-cell lymphoma (MCL), and Waldenström’s macroglobulinemia. Contrasting with this, a more heterogeneous pattern of expression of BCL-2 is reported among other hematological neoplasms, such as diffuse large B-cell lymphoma (DLBCL), for which certain subtypes present low levels of this molecule; and multiple myeloma (MM), in which BCL-2 expression is especially elevated in patients harboring t(11;14) [8].

Increased levels of BCL-2 can be driven by diverse mechanisms, including chromosomal translocations, gene amplification, and downregulation/deletion of genes encoding microRNAs (miRNAs) involved in BCL-2 RNA degradation [14]. In CLL, the most common leukemia in humans, overexpression of BCL-2 originates from the loss of repression by miRNA 15/16 [34]. Consistent with this, repression of BCL-2 at the post-transcriptional level by the microRNAs miR-15a and miR-16-1 was shown to induce apoptosis in a leukemic cell line model [34].

Cancer cells are found to be frequently primed for apoptosis [17]. Furthermore, it seems that a correlation exists between the level of mitochondrial priming and the degree of chemosensitivity. In hematological malignancies such as MM, acute myelogenous leukemia (AML), and acute lymphoblastic leukemia (ALL), a correlation was found between clinical responses to cytotoxic chemotherapy and mitochondria priming state prior to treatment; better responses to treatment were observed for patients’ cells with higher pretreatment mitochondrial propensity to undergo apoptosis [35, 36].

The recognition that BH3-only proteins are key initiators of apoptosis and that BCL-2 and other BCL-2 family members play an important role in cancer development and resistance to treatment prompted the development of drugs that mimic the action of the BH3 domain as they are able to restore apoptosis through the binding to one or more BCL-2 family members [9].

## BCL-2 inhibition by BH3 mimetics

A new class of anticancer drugs has emerged with the development of BH3-mimetic agents. This novel class of compounds is designed to selectively kill cancer cells by targeting the mechanism involved in their survival. These agents induce apoptosis by mimicking the activity of natural antagonists of BCL-2 and other related proteins [6, 9]. According to the criteria defined by Lessene and colleagues, true BH3-mimetics comprise compounds that fulfill the following characteristics: (1) biological activity dependent on BAX/BAK; (2) the compound must bind with high affinity (nanomolar levels) to at least one BCL-2 family member; (3) the killing activity of the compound must correlate with cellular levels of the target BCL-2 family members; and (4) the compound should affect biomarkers in animal models [6].

## BH3-mimetics that target BCL-2

### ABT-737

ABT-737, developed by Abbott Laboratories (North Chicago, IL, USA), is considered the prototype of BH3 mimetics as it was the ‘first-in-class’ compound developed to mimic the function of BH3-only-proteins. Discovered using a high-throughput nuclear magnetic resonance-based screening method to identify small molecules that bind to the BH3-binding groove of BCL-x_L_, ABT-737 binds with a much higher affinity (< 1 nmol/L) than previous compounds to anti-apoptotic proteins BCL-2, BCL-X_L_ and BCL-w, blocking their function [37]. On the other hand, it binds poorly to the other antiapoptotic BCL-2 family members sharing less similarity to BCL-X_L_, such as BCL-B, MCL-1, and A1 [38].

Single-agent cytotoxicity of ABT-737 was observed both against established cell lines derived from lymphoid malignancies and in ex vivo assays using primary cells derived from patients with FL and CLL [38]. Furthermore, ABT-737 was reported to synergize with radiation and chemotherapeutic drugs such as etoposide, doxorubicin, and cisplatin in various tumor cell lines [38]. Additional in vitro studies confirmed that ABT-737 displays single agent activity against multiple cancer cell lineages, including MM [39] and AML [40], without affecting normal hematopoietic cells [39, 40]. Moreover, it potentiates several chemotherapeutic and other anti-cancer drugs in AML, MM and chronic myelogenous leukemia (CML), and other settings [38–42]. In a cell line derived from CML (K562), co-treatment with ABT-737 circumvented the resistance to imatinib related to BCL-2 upregulation or loss of BIM or BAD [41].

Although treatment with ABT-737 induces massive apoptosis in multiple tumor cell lines derived from solid tumors, leukemia, and lymphoma, its killing activity is heterogeneous and resistance has been attributed to other members of BCL-2 family that do not interact with ABT-737, especially MCL-1 [14, 43, 44]. In AML, activity of ABT-747 was shown to be abrogated by BCL-2 phosphorylation or elevated MCL-1 [40].

Despite the favorable preclinical results, the development of ABT-737 for clinical use was constrained by its low aqueous solubility and the fact that this compound is not orally bioavailable [45]. To overcome these limitations and given the potential of this therapeutic strategy, researches invested in generating new compounds that would carry the pharmacologic properties of ABT-737, but would be orally bioavailable to optimize utility as single agent or within a combined regimen with other chemotherapeutics [42].

### Navitoclax (ABT-263)

Navitoclax, a potent and selective inhibitor of BCL-2, is the second generation, orally bioavailable form of ABT-737. Like its predecessor, navitoclax interacts with high affinity and abrogates BCL-2, BCL-X_L_, and BCL-w, but has no activity against A1 and MCL-1. Pharmacokinetically, it exhibited low plasma clearance values and low volumes of distribution in four distinct species [42].

Navitoclax showed in vitro activity against a broad panel of tumor cell lines both as single agent and in combination with chemotherapy. In in vivo experiments, treatment with this inhibitor induced rapid and complete tumor responses in multiple xenograft models developed using small-cell lung cancer and hematologic cell lines, with responses lasting several weeks in some models. Moreover, in B-cell malignant xenograft models, co-treatment with navitoclax significantly improved the efficacy of numerous approved anti-cancer agents. Navitoclax potentiated the activity of rituximab in the B-cell lymphoma flank xenograft model, of modified R-CHOP regimen in a flank xenograft model of MCL, and of bortezomib in a MM model [42]. Like ABT-737, navitoclax induced a rapid but reversible thrombocytopenia, reflecting apoptosis of circulating platelets as the result of blockage of BCL-2 family proteins, especially BCL-x_L_. Similarly to its predecessor, apoptosis induction by navitoclax also depends on neutralization of MCL-1 [42].

Regarding its mechanism of action, navitoclax-mediated cytotoxicity involves the impairment of interactions between BCL-2/BCL-X_L_ and pro-apoptotic members of the BCL-2 family (such as BIM), BAX translocation, and subsequent release of cytochrome c and apoptosis. This process was shown to be caspase-dependent [42].

In vivo studies conducted by Ackel et al. showed that inhibition of BCL-2 family proteins by navitoclax potentiates the response of B-cell lymphoma and MM models to distinct standard chemotherapeutic drugs and regimens such as etoposide, rituximab, bortezomib, cyclophosphamide, VAP (vincristine, adriamycin [doxorubicin], and prednisone), CHOP (cyclophosphamide, doxorubicin, vincristine, and prednisone), and R-CHOP (rituximab plus CHOP) [46].

Based on the promising results obtained in vitro and in vivo, clinical studies were designed. The antitumor activity and safety of two schedules of navitoclax were evaluated in a dose-escalation phase I trial conducted in patients with relapsed or refractory lymphoid malignancies. Responses were detected in distinct tumor types, with partial response being observed in 10 of 46 evaluable patients [47]. Despite the initial excitement with this agent, clinical use of navitoclax is restrained by its *on target*-effect on platelets. Thrombocytopenia was identified as the dose-limiting toxicity (DLT) of navitoclax in patients and is associated with the inhibition of BCL-X_L_, as previous studies showed that survival of platelets largely depend on BCL-X_L_ expression [47].

Daily navitoclax was also evaluated in patients with relapsed or refractory CLL. Navitoclax showed anti-leukemic activity against CLL, but a more heterogeneous pattern of response was observed when compared with what was predicted by in vitro data. Partial response was achieved in 35% of patients who received a daily dose of at least 110 mg. Acute and transient reductions in platelet counts were also reported. Dose reductions were necessary in five cases, and were mostly related to severe thrombocytopenia in cycles 1 or 2. Occurrence of grade 4 thrombocytopenia during the first cycle was decreased by the application of a lead-in dosing of daily 100 mg for 7 days followed by dose escalation [48]. Diarrhea, nausea, vomiting, fatigue, thrombocytopenia, and neutropenia were the most frequent AEs. Thrombocytopenia was the main DLT and was dose-related [48].

Navitoclax has been evaluated mostly in combination with other drugs, especially in solid tumor [22]. Among the hematological neoplasms, navitoclax was evaluated in combination with rituximab in phases I and II studies conducted in patients with relapsed or refractory CD20^+^ lymphoid malignancies [49] and patients with B-cell CLL with no prior treatment [50], respectively. Combination of navitoclax and rituximab was well tolerated and showed an important synergistic effect in both settings [49, 50].

### Venetoclax

#### Pre-clinical development

The development of selective inhibitors for BCL-2 and BCL-X_L_ is limited by the high degree of similarity shared by their BH3 domain [51]. Using reverse engineering, a new compound was developed to overcome the unfavorable effect of navitoclax on platelets as the consequence of BCL-X_L_ inhibition, while keeping its anti-tumor activity [52]. Venetoclax is a potent and selective inhibitor of the BCL-2 protein that has demonstrated clinical efficacy in several hematological malignancies [8, 53]. Contrasting with navitoclax, which targets BCL-2 and BCL-X_L_, venetoclax has a distinct mode of action as it binds and neutralizes BCL-2 with subnanomolar affinity (*Ki* < 0.010 nM), while interacting only weakly with BCL-X_L_ and BCL-W [52]. By sparing BCL-X_L_, it exerts little effect on platelet numbers. In preclinical studies, this orally bioavailable inhibitor showed cell-killing activity against a variety of cell lines, including cell lines derived from ALL, NHL, and AML. When investigated in xenografts models using hematological tumors, venetoclax promoted tumor growth inhibition in a dose-dependent fashion. For those models in which venetoclax had little effect as single-agent, improved efficacy was achieved with the combination with other drugs [52].

Additional in vitro studies demonstrated that normal and malignant human peripheral blood B-cells were highly susceptible to venetoclax, contrasting with T-cells and granulocytes that were less sensitive [54]. The lower hematological toxicity of venetoclax compared with navitoclax was also confirmed by in vitro and in vivo studies [54]. Regarding its mechanism of action, venetoclax-induced cytotoxicity in lymphoid cells significantly relies on the activation of BAX and BAK [54]. Experiments using Z-VAD, a wide-spectrum caspase inhibitor, revealed that cell-killing mediated by venetoclax is dependent on caspase activation [52]. Moreover, BIM seems to be the main initiator of apoptosis induced by this BH3-mimetic, as cell sensitivity to venetoclax is drastically abrogated in cells lacking this molecule. However, different levels of resistance due to the absence of BIM were observed across distinct subsets of lymphoid cells [54].

The efficacy of venetoclax was also investigated in childhood ALL [55] based on its better tolerability profile and the observation that ALL xenografts were sensitive to inhibition of BCL-2 by navitoclax [42]. A strong correlation between poor response to venetoclax and the expression of BCL-X_L_ was observed. Unlike CLL cells that largely depend on the expression of BCL-2 alone, killing of most ALL xenografts required simultaneous inhibition of BCL-2 and BCL-X_L,_ explaining the contrasting results obtained with venetoclax and navitoclax. The only exception was the xenografts from mixed lineage leukemia-rearranged infant ALL, in which venetoclax had a similar effect of navitoclax, and may represent an interesting subgroup for clinical trials with venetoclax [55].

#### Clinical studies with venetoclax in monotherapy in CLL

Based on the promising preclinical results, a first-in-human phase 1 dose-escalation study was conducted to investigate the safety and efficacy of daily oral venetoclax in patients with relapsed or refractory CLL or small lymphocytic lymphoma (SLL) [56]. A total of 116 patients were included considering both the dose-escalation and the expansion cohorts. At study admission, nearly 90% of the patients had genetic or clinical characteristics known to be associated with adverse prognosis, including chromosome 17p deletions, chromosome 11q deletions, unmutated immunoglobulin heavy-chain variable region, resistance to fludarabine, and bulky adenopathy. In the majority of the cases, at least two of these features were present. Regarding exposure to previous treatments, most patients had received multiple previous therapies (median of three, ranging from 1 to 11). Activity was detected at all dose levels tested, with impressive reduction of tumor burden in all tissue compartments. Overall response rates (ORRs) in the dose-escalation and expansion cohorts were of 77 and 82%, respectively. Notably, ORRs higher than 70% were also reported for patients carrying the deletion 17p and among patients with unmutated IGHV or resistance to fludarabine, with complete response (CR) rates > 15% in all cases [56]. Patients in the dose-escalation cohort had a median progression-free survival (PFS) of 25 months (95% confidence interval [CI], 17 to 30). The median PFS was 16 months among CLL patients with deletion 17p. Due to the short follow-up at the time of publication, PFS could not be estimated for the expansion cohort. CRs were associated with longer duration of response. An overall survival rate of 84% was estimated at 2 years [56].

Venetoclax had a favorable safety profile. In the dose escalation cohort, tumor lysis syndrome was detected in 18% of the patients and represented the most relevant toxicity. Based on this, a stepwise ramp-up strategy up to 400 mg was used in the expansion cohort, together with prophylaxis and treatment for cases identified as at risk for developing this adverse event, which did not occur in the expansion cohort. Diarrhea, upper respiratory tract infection, nausea, and neutropenia were the most frequent adverse events, occurring in nearly half of the patients. Neutropenia was the most common grade 3 or 4 toxicity, with 33 patients having grade 4 neutropenia. Febrile neutropenia was reported in 6% of patients and was the most common serious adverse event. In this study, a maximum tolerated dose was not identified, and the dose of 400 mg per day used for the expansion cohort was decided based on the efficacy and safety data obtained in the dose-escalation phase [56].

The impressive high response rates reported for CLL patients with deletion 17p treated with venetoclax were confirmed in a subsequent multicentric phase 2 trial [10]. A total of 107 patients with relapsed/refractory CLL with deletion 17p and who had already undergone at least one type of treatment were included. After a weekly dose ramp-up schedule, patients received continuous venetoclax at 400 mg/day. Analysis of the ORR assessed by independent review, the study primary endpoint, revealed that venetoclax led to a response rate of 79.4% (95% CI, 70.5 to 86.6). Consistent with the phase I study, high response rates were obtained irrespective to the presence of other poor-prognosis features [8, 10]. Regarding safety, neutropenia (40%) followed by infection (20%), anemia (18%), and thrombocytopenia (15%) were the most common grade 3 or 4 events. Grade 4 neutropenia was detected in 23% of patients and responded to dose reductions or the use of granulocyte colony-stimulating factors. Fifty-nine patients had serious adverse events, with febrile neutropenia being registered in five patients [10]. Based on these promising results and to accelerate its development, venetoclax was granted the status of ‘breakthrough therapy designation’ by the FDA. In April 2016, this inhibitor was approved by the FDA for the treatment of patients with CLL who have 17p deletion and have received at least one prior therapy. In December 2016, venetoclax monotherapy received the approval of the European Medicines Agency for the treatment of CLL in the presence of 17p deletion or TP53 mutation in adult patients who are unsuitable for or have failed a B-cell receptor pathway inhibitor (BCRi); and for CLL in the absence of 17p deletion or TP53 mutation in adult patients who have failed both chemoimmunotherapy and a BCRi [57].

Venetoclax as single agent is being evaluated in patients with CLL who relapsed after or are refractory to ibrutinib (Arm A) or idelalisib (Arm B), two BCRis. Results of the phase 2 M14-032 trial (NCT02141282) demonstrated that monotherapy with venetoclax is well tolerated and effective in this setting, promoting high rates of durable response (ORR of 70 and 62% in arm A and B, respectively, as assessed by an Independent Review Committee) and of MRD-negativity [58]. A post hoc subgroup analysis of 28 patients who had received > 1 prior BCRi out of the 127 enrolled in this study showed that venetoclax safety profile in this subgroup was consistent to what was expected in relapsed/refractory CLL patients, and, although response rates were lower than in other settings, venetoclax was shown to be active in this subgroup [59]. A more recent publication of this trial reported promising results (ORR of 67%; median time to first response 2.5 months) and a well-tolerated safety profile for monotherapy with venetoclax when data of 36 patients with CLL refractory to or who relapsed after idelalisib were analyzed. Hematologic events accounted for most grade 3 or 4 adverse events reported, and tumor lysis syndrome was not observed [60]. Similarly, an interim analysis of the 91 patients (43 from the main cohort and 48 in the expansion cohort) whose disease progressed during or after discontinuation of ibrutinib, revealed an ORR of 65% and a safety profile within the expected as neutropenia, thrombocytopenia, and anemia were the most common treatment-emergent grade 3 or 4 toxicities [61].

In a pooled analysis of three trials, relapsed/refractory CLL patients with fewer number of prior lines of treatment and less disease bulk were reported to benefit the most from the venetoclax monotherapy when outcomes as CR rate, PFS, and OS were analyzed, suggesting that favorable outcomes are more likely to occur with the earlier introduction of venetoclax [62].

#### Clinical studies with venetoclax in monotherapy in other hematological malignancies

The safety and activity of venetoclax in monotherapy have also been evaluated in other clinical settings, such as relapsed or refractory lymphoma and MM [8, 63, 64].

A total of 106 patients were enrolled in a phase I dose-escalation study of venetoclax in patients with relapsed/refractory NHL. Among the different subtypes of NHL included in the dose-escalation cohort, MCL, DLBCL, and FL were the most common. Overall, venetoclax showed a tolerable safety profile. Treatment-emergent adverse events were reported by nearly all cases (97%) and were mostly grade 1 to 2 in severity. A total of 59 (56%) patients reported grade 3 to 4 events, with hematologic toxicities being the most frequent. Regarding its efficacy, an ORR of 44% was achieved; however, the activity of venetoclax as monotherapy varied among NHL subtypes. Among patients with DLBCL, the ORR was 18%, and in many patients, response was not sustained. Clinical results in FL and MCL patients were considered favorable, as the ORRs were 38 and 75%, responses had longer duration, and median PFS was 11 and 14 months, respectively. In all settings, it is possible that patients would benefit from the use of venetoclax in combination therapies to improve response intensity and duration [64].

In heavily pretreated (median of 5 prior therapies) relapsed/refractory MM, monotherapy with venetoclax also showed a tolerable safety profile. As expected, most commonly reported adverse events were mild to moderate gastrointestinal toxicities, including nausea, diarrhea, and vomiting. Two patients experienced DLTs of epigastric pain and nausea with abdominal pain. Overall response was achieved by 21% (14/66) of the patients, while 15% (10/66) achieved very good partial response (VGPR) or better. Higher response rates were reported among patients harboring t(11;14). Efficacy data of this phase I study with dose-escalation and expansion cohorts suggest that this strategy may be especially interesting in patients with t(11;14), whose disease is marked by low expression of BCL-X_L_ and high BCL-2 [63].

The safety and efficacy of venetoclax in monotherapy are being investigated in a number of clinical trials (Table 1).

**Table 1:** Clinical trials investigating venetoclax as single agent in hematological malignancies

Disease(s)	Study phase	Estimated* or actual** enrollment	Register number in clinical trials.gov
NHL (non CLL/SLL/MCL)	I	11**	NCT01969695
R/R CLL after BCRis (ibrutinib or idelalisib)	II	127**	NCT02141282
R/R CLL/SLL and NHL	I	211*	NCT01328626
R/R AML or untreated AML unfit for intensive therapy	II	32**	NCT01994837
R/R or untreated CLL with 17p deletion	II	158**	NCT01889186
R/R CLL	III	250*	NCT02756611
R/R CLL with 17p deletion	II	70*	NCT02966756
R/R CLL	III	200*	NCT02980731
Waldenström Macroglobulinemia	II	33**	NCT02677324

*AML* acute myelogenous leukemia, *BCRi* B-cell receptor signaling pathway inhibitors, *CLL* chronic lymphocytic lymphoma, *NHL* non-Hodgkin lymphoma, *R/R* relapsed/refractory, *SLL* small lymphocytic lymphoma, *V* venetoclax

Results retrieved from the Clinical Trials database (www.clinicaltrial.gov), accessed at 20 Feb 2018 *Estimated enrollment at the time of Clinical Trials database access. **Actual enrollment at the time of Clinical Trials database access

The following studies were not included in this table: study with status “withdrawn” (NCT02095574); studies on myelodysplasic syndromes, and observational studies (NCT03415035 and NCT03342144)

In addition to interventional trials, prospective observational studies (NCT03415035 and NCT03342144) are being conducted to assess venetoclax effectiveness regarding best response and response duration, survival endpoints, patient-reported outcomes in patients with CLL, and parameters related to treatment costs under real-life settings. In a real-world study, achievement of deep response in second-line therapy was found to be associated with improved long-term outcomes such as PFS and OS among patients with relapsed/refractory CLL [65].

#### Venetoclax in combination with other anticancer drugs

Besides its use as a single agent, venetoclax is being combined with several classes of agents, including DNA damaging chemotherapy, anti-CD20 antibodies (rituximab and obinutuzumab), proteasome inhibitors (bortezomib and carfilzomib), hypomethylating agents (azacitidine or decitabine), kinase inhibitors (sunitinib, ibrutinib, idelalisib, dinaciclib, cobimetinib), Mdm2 inhibitor (idasanutlin), and inhibitors of anti-apoptotic molecules Bcl-X_L_ and Mcl-1, to improve its efficacy and overcome resistance [8, 66]. The rational for these combinations is that, contrasting with CLL, FL, MCL, and Waldenström macroglobulinemia, which are marked by high levels of BCL-2 and malignant cells largely depend on this molecule for survival/resistance to death, in other hematological malignancies (including DLBCL, ALL, MM, AML, and CML), this anti-apoptotic molecule exhibits a more varied pattern of expression and its contribution to cell resistance to death is not ubiquitous. For example, resistance to venetoclax has been reported to rely on the overexpression of BCL-X_L_ or/and MCL-1 [66].

In these cases, the sole inhibition of BCL-2 is not sufficient to achieve important results in clinical outcomes. Therefore, different combinations are being investigated in these clinical settings, and the drugs combined with venetoclax are chosen based on the molecular pathways that are altered in the particular malignancy.

Most studies on combinations of venetoclax and other agents are still ongoing. Table 2 presents selected planned studies.

**Table 2:** Ongoing clinical trials investigating venetoclax in combination with other agents for the treatment of hematological malignancies

Intervention	Disease	Study phase(s)	Estimated* or actual** enrollment	Register number in clinical trials.gov
V + ibrutinib + obinutuzumab	MCL	I/II	48*	NCT02558816
V + ibrutinib	MCL	II	24*	NCT02471391
V + ibrutinib	R/R MCL	I	28*	NCT02419560
V + low-dose cytarabine	Treatment-naïve AML	I/II	94**	NCT02287233
V + rituximab	Relapsed CLL and SLL	I	49**	NCT01682616
V + bortezomib + dexamethasone	R/R MM	I	66**	NCT01794507
V + bendamustine + obinutuzumab	CLL	II	66**	NCT02401503
V + dexamethasone	R/R MM t(11;14)-positive MM	II	166*	NCT01794520
V + bendamustine/rituximab	R/R NHL	I	60**	NCT01594229
V + decitabine or azacitidine	AML	I	260*	NCT02203773
V + obinutuzumab	Untreated or R/R CLL	I	82**	NCT01685892
V + rituximab or obinutuzumab and standard doses of CHOP	NHL	I/II	267**	NCT02055820
V + rituximab vs bendamustine + rituximab	Relapsed/resistant CLL	III	389**	NCT02005471
V + bendamustine + rituximab vs BR alone	R/R FL	II	164**	NCT02187861
V + bortezomib and dexamethasone	R/R MM	III	291**	NCT02755597
V + bendamustine + rituximab or bendamustine and obinutuzumab	R/R or untreated CLL	I	84**	NCT01671904
V + obinutuzumab + ibrutinib	R/R or untreated CLL	I/II	63**	NCT02427451
V + obinutuzumab + vs obinutuzumab + chlorambucil	CLL with coexisting medical conditions	III	445**	NCT02242942
V + azacitidine vs placebo + azacitidine	Treatment-naïve AML	III	400*	NCT02993523
V + obinutuzumab	R/R DLBCL	II	21*	NCT02987400
V + carfilzomib + dexamethasone	R/R MM	II	40*	NCT02899052
V + ibrutinib	R/R FL	I/II	41*	NCT02956382
V + ibrutinib	CLL; SLL	II	160*	NCT02756897
V + obinutuzumab	Untreated FL	I	25*	NCT02877550
V + lenalidomide + obinutuzumab	R/R NHL	I	60*	NCT02992522
V + decitabine	R/R AML	II	160*	NCT03404193
V + standard chemotherapy	ALL	I	22*	NCT03319901
V with or without chemotherapy	Pediatric and young adult R/R malignancies (ALL, AML, NHL, and other non-hematological tumors)	I	135*	NCT03236857
V + ibrutinib	CLL/SLL with progressive disease on ibrutinib	I	24*	NCT03422393
V + obinutuzumab and bendamustine	FL	II	56*	NCT03113422
V + dose-adjusted EPOCH-R	Richter’s syndrome	II	20*	NCT03054896
V + navitoclax + chemotherapy	R/R ALL	I	42*	NCT03181126
V + ibrutinib	High-risk CLL; SLL	II	45*	NCT03128879
V + obinutuzumab, after different debulking regimens	Untreated CLL		100*	NCT03406156
V + ublituximab + umbralisib (TGR-1202)	R/R CLL; SLL	I/II	30*	NCT03379051
V + TAK-659	R/R NHL	I	53*	NCT03357627
V + R-ICE	R/R DLBCL	I	18*	NCT03064867
V + chemotherapy (FLAG-IDA)	AML	I/II	56*	NCT03214562
V + low-dose cytarabine vs low-dose cytarabine	Untreated AML	III	175*	NCT03069352
V + cobimetinib vs cobimetinib vs V + cobimetinib + atezolizumab	R/R MM	I/II	72*	NCT03312530
V + rituximab vs bendamustine + rituximab	R/R CLL	III	389**	NCT02005471
V + bendamustine, rituximab, and ibrutinib	R/R MCL	Early phase I	18*	NCT03295240
V + ibrutinib	R/R CLL/SLL	II	20*	NCT03045328
V + ibrutinib vs ibrutinib	MCL	III	287*	NCT03112174
V + ibrutinib + obinutuzumab	CLL patients with TP53 deletion (17p-) and/or mutation	II	40*	NCT02758665
V + ketoconazole	R/R NHL	I	12**	NCT01969669
V + atezolizumab + obinutuzumab	R/R FL, R/R agressive DLBCL, R/R indolent NHL	II	138*	NCT03276468
V + rituximab	R/R CLL; SLL	I	49**	NCT01682616
V + ixazomib citrate + dexamethasone	R/R MM	I/II	71*	NCT03399539
MOR00208 + V vs MOR00208 + idelalisib	R/R CLL/SLL Patients pretreated with BTKi	II	24*	NCT02639910
V + cobimetinib and V + idasanutlin	RR AML not eligible for cytotoxic therapy	I/II	140*	NCT02670044
V + DA-EPOCH-R	Aggressive B-cell lymphomas (including DLBCL; HGBCL, TiNHL, and unclassifiable with intermediate features between DLBCL and BL)	I	18*	NCT03036904
V + ibrutinib	R/R CLL with or without TP53 aberrations	II	230*	NCT03226301
V + ibrutinib	Treatment naïve CLL/SLL	II	289*	NCT02910583
V + RO6870810, with or without rituximab	R/R DLBCL	I	94*	NCT03255096
V + chemotherapy	R/R AML or acute leukemia of ambiguous lineage	I	54*	NCT03194932
V or V + azatidine or V + rituximab	AML, CLL, MM, NHL, SLL	I/II	37*	NCT02265731***
V + ibrutinib and rituximab	R/R DLBCL	I	30*	NCT03136497
Obinutuzumab + idasanutlin and V or rituximab + idasanutlin and V	R/R FL or R/R DLCBL	I/II	140*	NCT03135262
V + daratumumab and dexamethasone (with and without bortezomib)	R/R MM	II	90*	NCT03314181
Obinutuzumab, rituximab, polatuzumab vedotin, and V	R/R FL or DLBCL	I	134*	NCT02611323
Standard chemoimmunotherapy vs rituximab + V vs obinutuzumab + V vs obinutuzumab + Ibrutinib + V	Fit patients with untreated CLL without del(17p) or TP53 mutation	III	920*	NCT02950051
V + ibrutinib + prednisone + obinutuzumab,+ Revlimid	R/R B-cell lymphoma	I	38*	NCT03223610

*AML* acute myelogenous leukemia; *BL* Burkitt lymphoma; *CLL* chronic lymphocytic lymphoma; *DLBCL* diffuse large B-cell lymphoma. *FL* follicular lymphoma; *FLAG-IDA* chemotherapy includes fludarabine, cytarabine, idarubicin, and filgrastim; *HGBCL* high-grade B-cell lymphoma; *MCL* mantle cell lymphoma; *MM* multiple myeloma; *NHL* non-Hodgkin lymphoma; *RICE* rituximab, ifosfamide, carboplatin, etoposide; *R/R* relapsed/refractory; *SLL* small lymphocytic lymphoma; *TiNHL* transformed indolent NHL; *V* venetoclax

Results retrieved from the Clinical Trials database (www.clinicaltrial.gov) accessed at 20 Feb 2018 *Estimated enrollment at the time of Clinical Trials database access.**Actual enrollment at the time of Clinical Trials database access.

The following studies were not included in this table: studies with status “withdrawn” (NCT03342678, NCT02640833, NCT01969682); studies on myelodysplasic syndromes, expanded access (NCT03123029), and post-marketing observational study (NCT03310190)

Actual: recruitment status completed or active, not recruiting

***This trial has four arms of treatment: Arm A: phase I of venetoclax in monotherapy for R/R NHL or MM; Arm B: phase I of venetoclax in monotherapy in CLL/SLL; Arm C: phase 1 of venetoclax with the addition of azacitidine in participants AML; and Arm D: phase 2 of venetoclax with the addition of rituximab in R/R CLL

##### Combinations for CLL

In patient with relapsed or refractory CLL, venetoclax has been combined with rituximab showing favorable efficacy results and a good safety profile [67]. Results from a phase 1b, dose escalation trial showed deep and durable responses and suggested benefit of retreatment. Overall and complete responses were achieved by 86 and 51% of the 49 patients enrolled in the study, respectively. The safety profile was considered acceptable [67].

Results from an interim analysis of the phase 3 MURANO study (NCT02005471) demonstrated that in patients with relapsed/refractory CLL, combination of venetoclax and rituximab was superior to bendamustine plus rituximab, improving responses and MRD-negativity rates and prolonging PFS. This positive effect on PFS was found to be consistent across subgroups, including among patients with and without del(17p) [68]. Promising efficacy results have also been reported for a sequential treatment with a debulking with bendamustine followed by obinutuzumab and venetoclax as induction and maintenance therapy in physically fit and unfit, treatment-naïve, and relapsed/refractory CLL patients [69].

A pooled analysis of data from 323 patients with relapsed or refractory CLL/SLL from three clinical trials evaluated the effect on PFS of venetoclax in monotherapy or co-administered with rituximab. Using a time-variant relative risk survival model, Freise et al. found that venetoclax affects PFS in a dose-dependent manner. Moreover, concomitant administration with 6 cycles rituximab provided a synergistic benefit with important prolongation of PFS median estimates (1.8 years [95% CI, 1.7–2.1] vs 3.9 years [95% CI, 2.8–5.6]) [70].

##### Combinations for MM

Positive results were also obtained for the combination of venetoclax, bortezomib, and dexamethasone in patients with relapsed/refractory MM who had received a median of three prior therapies. Data from the phase Ib study conducted by Moreau et al. suggest that this combination is safe and active, with an ORR of 67% and higher response rates obtained in patients with no prior treatment [71]. VGPR or better were achieved by 28 of 66 patients (42%); 10 patients achieved CR. Median duration of response was 9.7 months, while median time to progression was 9.5 months. Higher response rates, more durable responses and longer time to progression were observed for patients who were non-refractory to bortezomib and who had received 1 to 3 prior lines. Higher levels of BCL2 were reported for patients who achieved partial response or better. Gastrointestinal toxicities of mild severity and grade 3–4 cytopenias were the most frequent adverse events [71].

A phase 1 study is evaluating venetoclax as single agent or in combination with dexamethasone in patients with relapsed/refractory, heavily pretreated MM. Analysis of 50 patients with t(11;14) MM treated in the study (30 with venetoclax and 20 with the combination) revealed that both regimens were well tolerated. Regarding efficacy outcomes, the combination with dexamethasone was associated with-higher ORR (65 vs 40%) than venetoclax monotherapy [72].

Primary plasma cell leukemia, the most aggressive form of MM, has a high prevalence of t(11;14). A very deep and impressively rapid hematologic response was achieved when a combination regimen incorporating venetoclax with daratumumab, bortezomib, and dexamethasone was used to treat a 55-year-old female patient with very refractory primary plasma cell leukemia with t(11;14) [73], reinforcing the importance of investigating new treatment options including BCL-2 inhibitors.

##### Combinations in AML

The combination of venetoclax and hypomethylating agents seems to be an attractive therapeutic strategy for AML. Recently, a subgroup analysis of the phase III DACO-016 trial suggested that the hypomethylating agent decitabine improves response rates and prolong PFS in elderly (≥ 65 years) patients with newly diagnosed with AML and with monosomal karyotype [74].

The safety and efficacy of venetoclax combined with azacitidine or decitabine is under investigation in a phase 1b study conducted in elderly patients (≥ 65 years at diagnosis) with previously untreated AML who were not eligible to for intensive induction therapy (NCT02203773) [75]. Although four patients died within the first dose of venetoclax due to sepsis, bacteremia, lung infection, and respiratory failure, a tolerable safety profile was otherwise observed. Thrombocytopenia (47%), febrile neutropenia (42%), and neutropenia (40%) were the most common grade 3–4 treatment-emergent adverse events reported. Tumor lysis syndrome was not reported. Preliminary efficacy results were favorable, with high rates of response. Complete remission or complete remission with incomplete marrow recovery was achieved by 61% of 57 patients; similar results were obtained in either combination. The dose of 400 mg of venetoclax was found to provide the best benefit-risk balance. A phase 3 trial (NCT02993523) combining this dose of venetoclax with azacitidine vs azacitdine plus placebo is ongoing in treatment-naïve AML patients not eligible for induction.

Promising results are likely to arise for AML patients from the new combinations of venetoclax with four drugs (midostaurin, enasidenib, CPX-351, and gemtuzumab ozogamicin) that received approval for AML by the FDA in 2017 [76].

##### Combinations in other hematological malignancies

Another group of patients that may benefit from the combination of ventoclax and rituximab is refractory cutaneous B-cell lymphoma (CBCL) patients, as upregulation of BCL-2 was suggested to be involved in treatment resistance to rituximab [77].

#### Additional in vitro and in vivo studies

The ex vivo sensitivity of leukemic cells derived from 73 AML patients was evaluated to search for biomarkers that could be used to predict response to BCL-2 blockage. Responses were more variable among AML samples than among samples derived from CLL patients, which are known to be highly sensitive to this therapeutic approach or compared with non-sensitive cells derived from healthy donors and used as controls. An important upregulation of specific HOXA and HOXB gene transcripts was found in AML cells highly sensitive to BCL-2-inhibitiors. Based on the strong correlation observed between response and HOX gene expression, the authors suggested that HOX gene expression pattern may be a useful marker to improve the selection of AML patients that are more prone to respond to BCL-2 inhibitor in clinical trials [78].

Blastic plasmacytoid dendritic cell neoplasm (BPDCN), a rare and aggressive hematologic malignancy with poor prognosis and for which no standardized therapeutic approach has been established, is another neoplasm that may potentially respond to venetoclax. Recently, Montero et al. showed that primary BPDCN cells are highly dependent on BCL-2 protein and sensitive to venetoclax. In addition, tumor regression with improved survival was achieved with venetoclax in an animal model using BPDCN patient-derived xenografts. Notably, significant disease responses were observed in two patients with relapsed/refractory BPDCN who received venetoclax off-label [79].

BCL2 and MYC are oncogenes commonly deregulated in lymphomas. DLBCL represents 30 to 40% of all NHL. It is a heterogenous disease clinically, morphologically, and molecularly. Based on studies on gene expression profile, important subgroups for prognosis were defined for DLBCL. The chromosomal translocation t(14;18)(q32;q21) is found in nearly 20% of DLBCL cases [80, 81]. In this setting, the presence of this chromosomal abnormality was shown to define a unique subset of DLBCL within the germinal center B-cell-like subgroup [80]. In approximately 5% of DLBCL, translocations t(14;18) and t(8;14) resulting in overexpression of BCL-2 and MYC, respectively, occur concurrently [82].

The importance of BCL-2 and MYC as therapeutic targets for DLBCL with concomitant overexpression of these two proteins was investigated by Sasaki et al. using two cell lines established from chemoresistant patients. Inhibition of BCL-2 by ABT-263 had a more potent cell-killing activity than blocking MYC using a specific inhibitor (10058-F4). Moreover, combination of ABT-263 with genotoxic agents had additive and/or synergistic effect on apoptosis promotion, whereas a more modest effect was observed with the association of the MYC inhibitor and genotoxic drugs. Notably, simultaneous inhibition of BCL-2 and MYC was more effective than either agent alone [83].

More recently, Boidol et al. published the first evidence of activity of venetoclax as single agent both ex vivo and in human in T-cell prolymphocytic leukemia [84].

## Conclusion

The association between BCL-2-family proteins and hematologic malignancies is well established. Consistent with this observation, drugs that mimic key modulators of apoptosis have emerged over the last years producing favorable outcomes. Inhibitors of BCL-2, such as venetoclax and navitoclax, were shown to selectively induce apoptosis in malignant cells and have been extensively investigated as single agents and in combination with other drugs in several malignancies, including acute leukemia, lymphomas, and solid tumors. Navitoclax was shown to be an important inhibitor in clinical subgroups in which the exclusive inhibition of BCL-2 is not sufficient. Important response rates were obtained with venetoclax even in CLL patients with disease features implicated with poor outcomes with chemoimmunotherapy. Results presented so far support that BH3-mimetics may be useful components in several treatment regimens given their synergism with chemotherapy drugs and other agents. As interactions between BH3-only proteins and anti-apoptotic members of the BCL-2 family occur in a selective manner, elimination of cancer cells may require the blockage of all or some anti-apoptotic BCL-2 proteins at the same time to induce death [3]. Several studies are now focused on the identification of expression signatures that could be used to predict sensitivity to the BCL-2 inhibitors and allow the identification of subgroups of patients that would benefit more from more selective or more promiscuous inhibitors.

## Abbreviations

ALL: Acute lymphoblastic leukemia
AML: Acute myelogenous leukemia
BCL-2: B-cell lymphoma-2
BCRi: B-cell receptor pathway inhibitor
BH: BCL-2 homology domain
BPDCN: Blastic plasmacytoid dendritic cell neoplasm
CBCL: Cutaneous B-cell lymphoma
CLL: Chronic lymphocytic leukemia
CR: Complete response
DLBCL: Diffuse large B-cell lymphoma
MM: Multiple myeloma
MOMP: Mytochondria outer membrane permeabilization
NHL: Non-Hodgkin lymphoma
ORR: Overall response rate
PFS: Progression free survival
R/R: Relapsed/refractory
SLL: Small lymphocytic lymphoma
VGPR: Very good partial response
